# Supplementary material for: Optimization of Blood Handling and Peripheral Blood Mononuclear Cell Cryopreservation of Low Cell Number Samples
Source: Int J Mol Sci. 2021 Aug 24;22(17):9129. doi: 10.3390/ijms22179129 (PMC8431655; doi:10.3390/ijms22179129)
Supplement: Supplementary file 1 [file ijms-22-09129-s001.zip › ijms-1317614-supplementary.pdf]

International Journal of
Molecular Sciences

MDPI

Supplementary material

Optimization of Blood Handling and Peripheral Blood Mononuclear Cell Cryopreservation of Low Cell Number Samples

Supplementary material

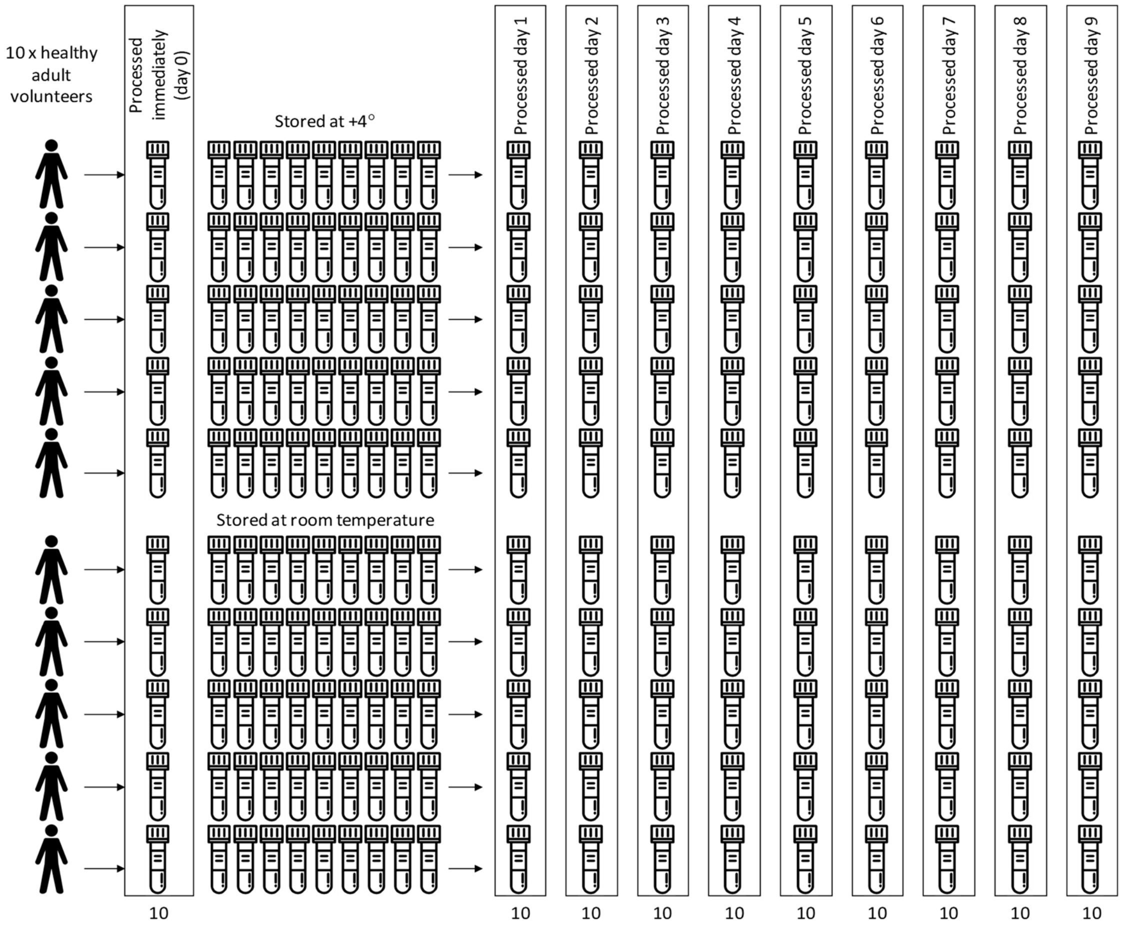

Figure S1. Sample processing in Experiment 1.

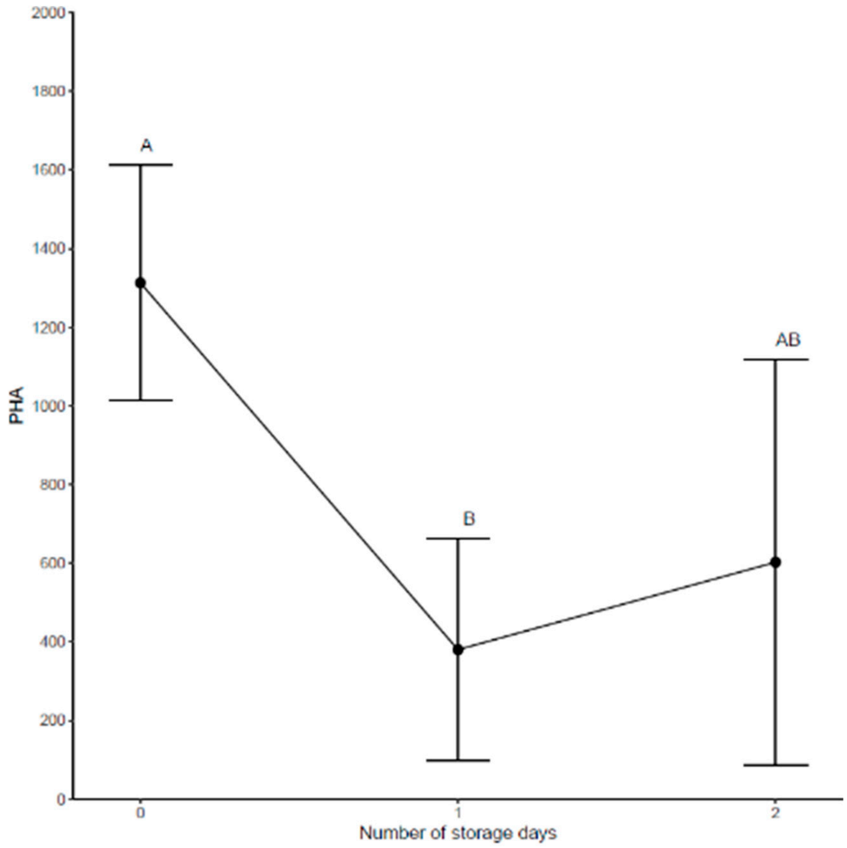

Figure S2. Impacts of delaying blood processing on PHA. Each point represents the predicted mean with 95% CI. Letters indicate groups (Tukey's test), where predicted means that are not significantly different are labelled with the same letter. No significant differences between 4°C and RT were found, therefore storage day means only are shown.

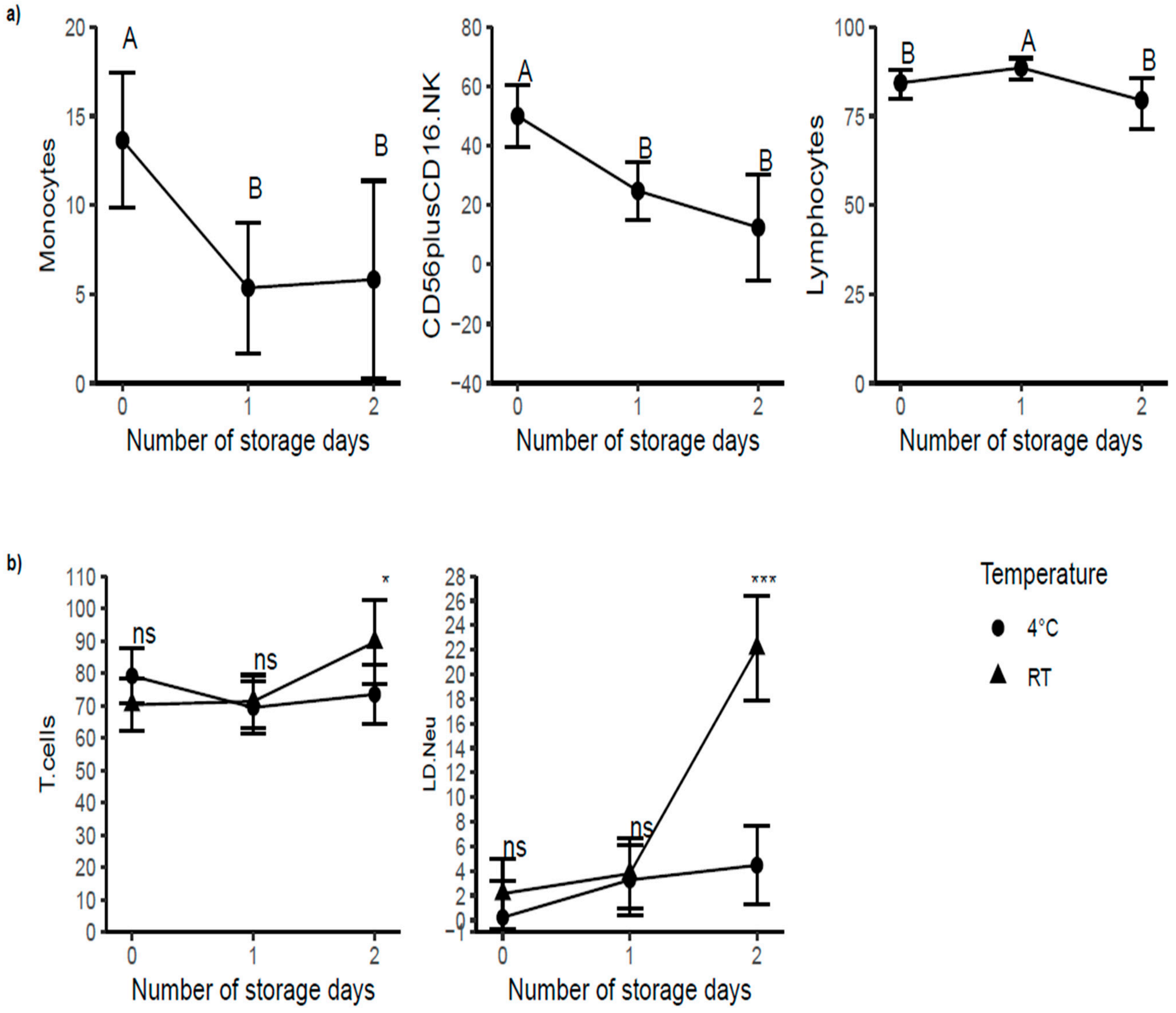

Figure S3. Impacts of delaying blood processing on immunophenotyping analysis regarding (a) NK cells, monocytes and lymphocytes. Letters indicate groups (Tukey's test), where predicted means that are not significantly different are labelled with the same letter. No significant differences between 4°C and RT were found, therefore storage day means only are shown. (b) T cells, low density neutrophile. Significance (LSD) is the difference between 4°C and RT at a particular day; where ns = not significant, *$p<0.05$, **$p<0.01$, ***$p<0.001$. Each point represents the predicted mean with 95% CI.

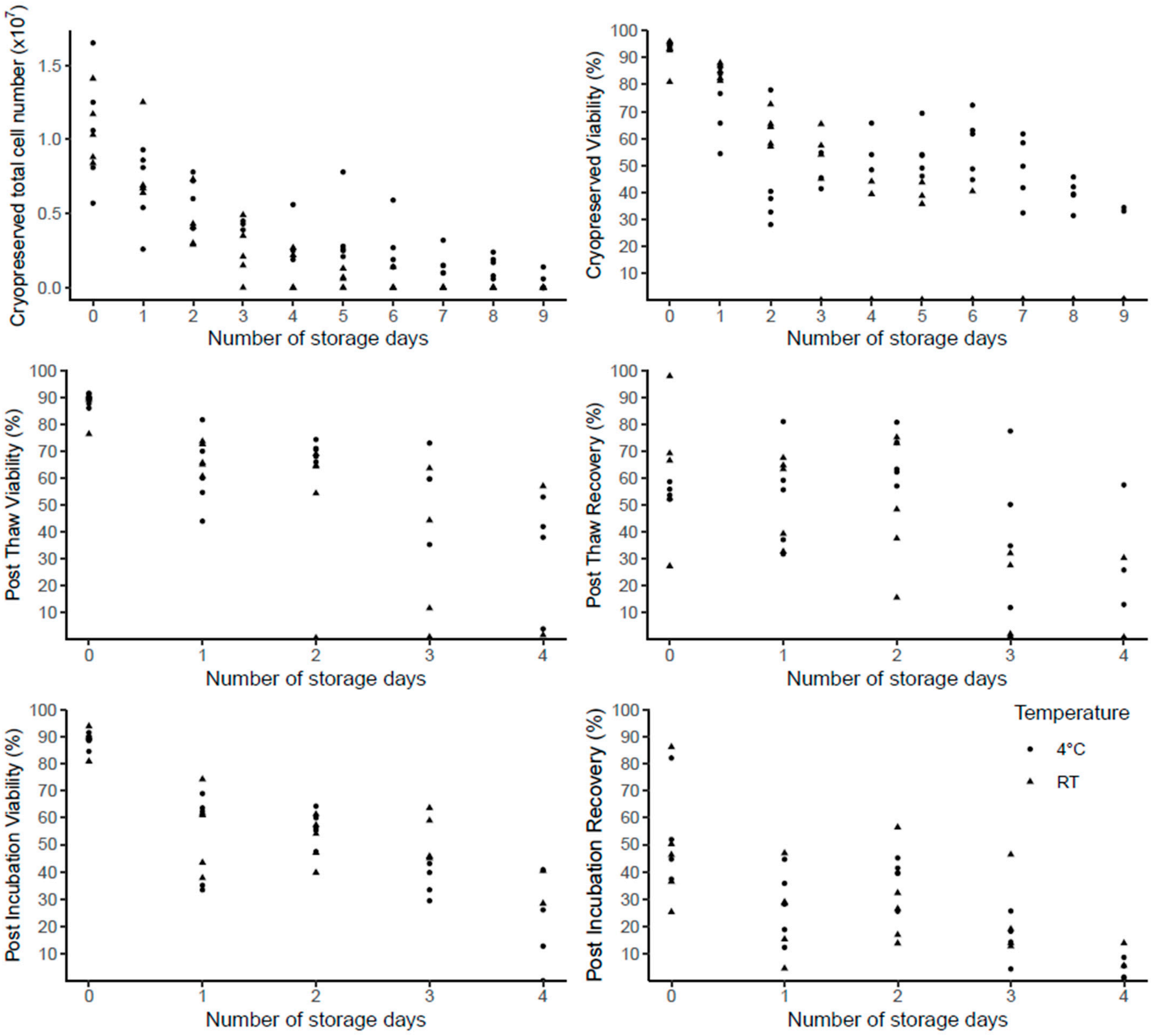

Figure S4. Experiment 1 - Impacts of delaying blood processing on PBMC quality in term of viability and recovery measured at storage, post thaw and post incubation raw data plots.

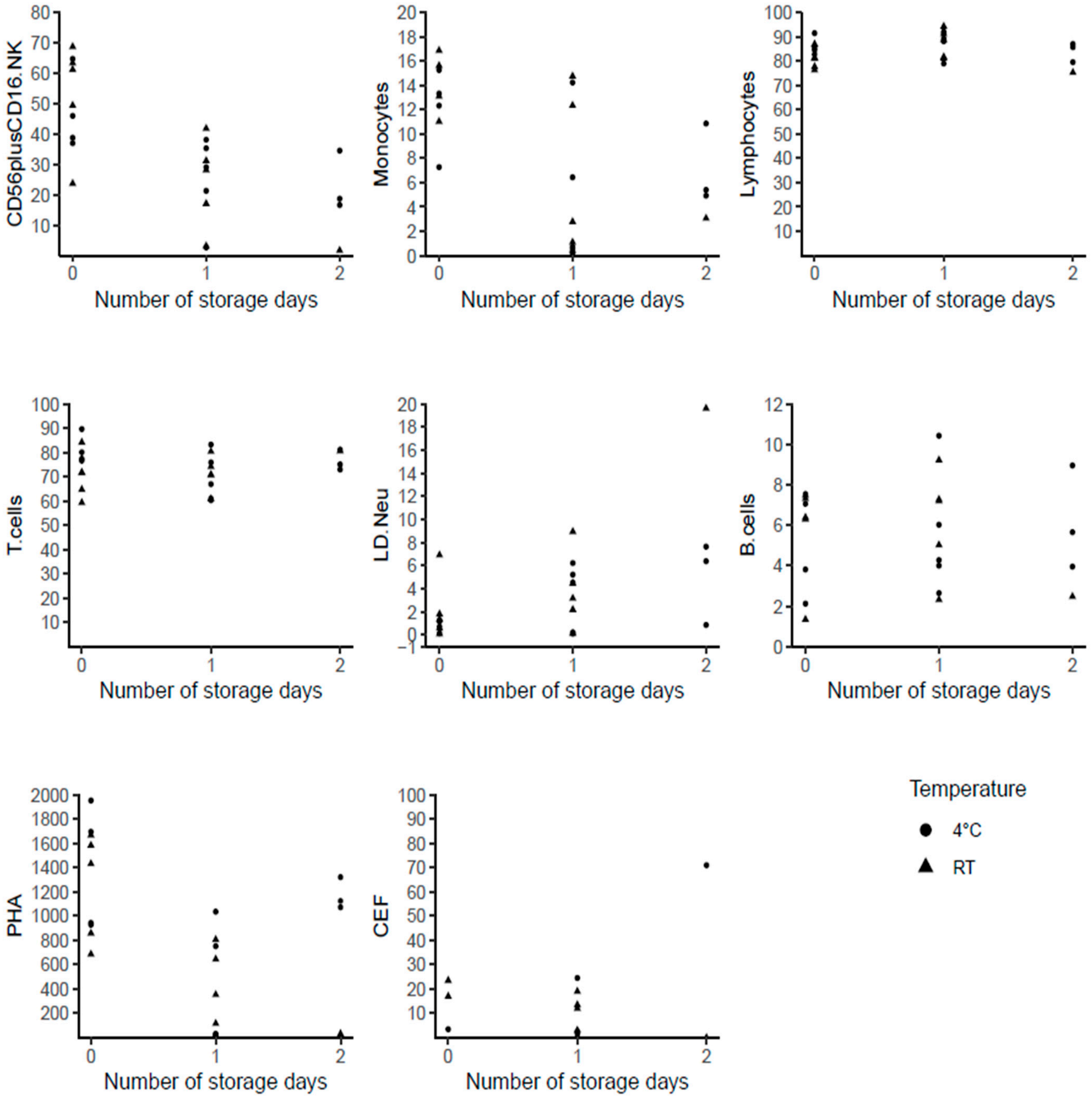

Figure S5. Experiment 1 - Impacts of delaying blood processing on PBMC quality in term of immunophenotyping and functional analysis raw data plots.

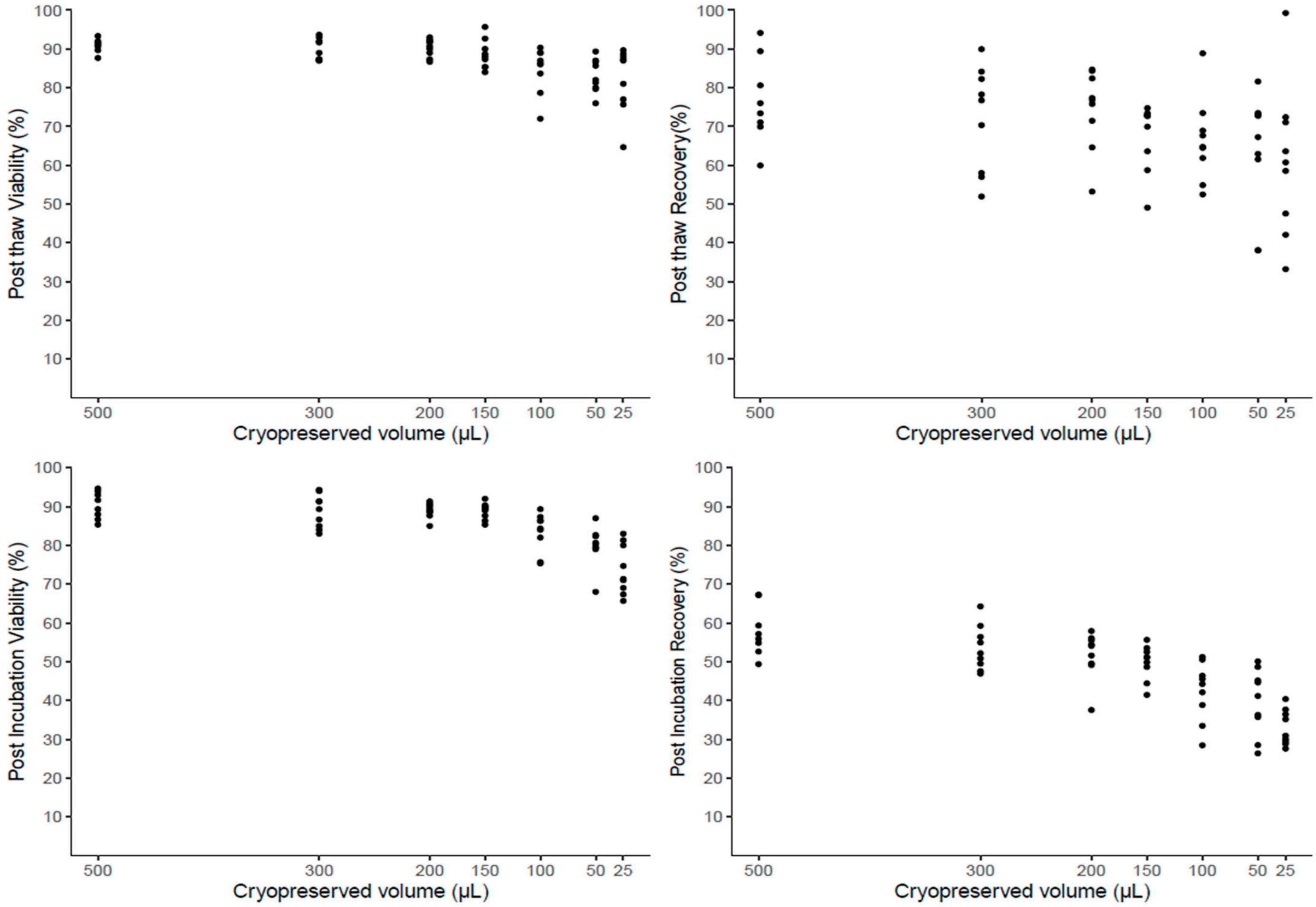

Figure S6. Experiment 2 – Impacts of cryopreserved cell volumes on PBMC quality raw data plots.

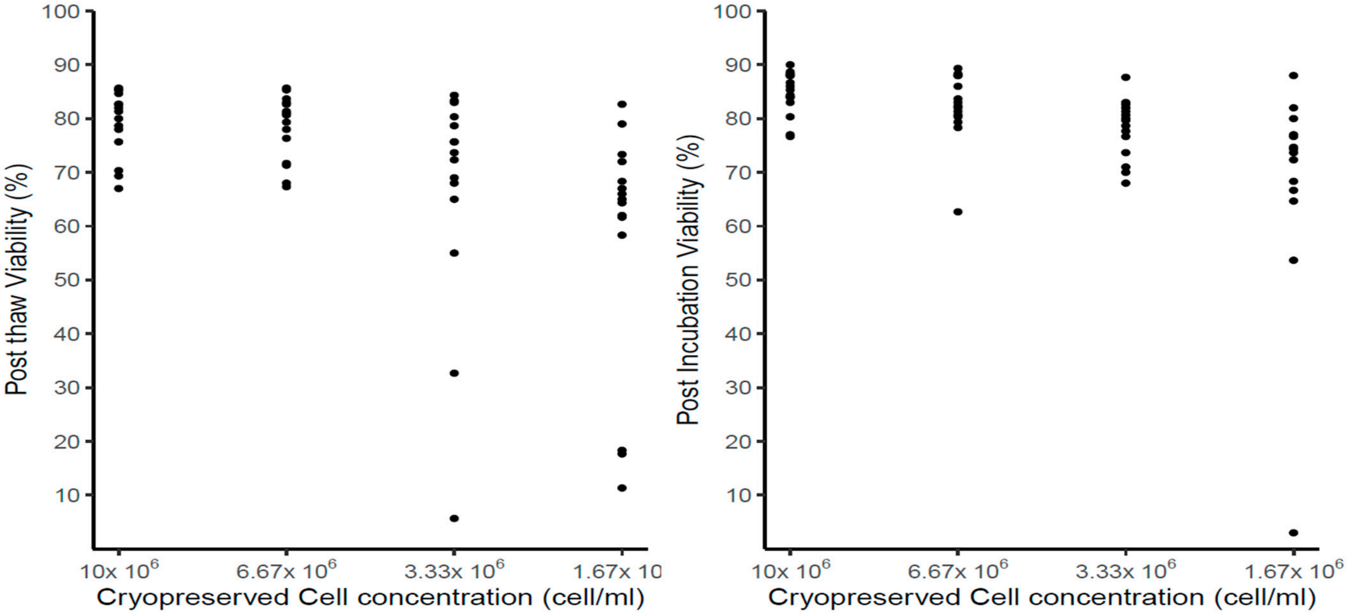

Figure S7. Experiment 3 – Impacts of cryopreserved cell concentrations on PBMC quality raw data plots.

Table S1. The comparison in post thaw viability and recovery measured at different days of storage.

Day	Post Thaw Viability (%)	Post Thaw Recovery (%)
0	88.72 (75.51, 95.25)	58.45 (44.89, 72.01)
1	65.48 (43.94, 82.11)	53.25 (40.26, 66.23)
2	53.55 (32.27, 73.62)	58.63 (45.65, 71.62)
3	34.66 (16.51, 58.74)	31.15 (17.01, 45.29)
4	19.06 (6.58, 44.05)	19.91 (3.31, 36.52)

Data are predicted means (95%CI). Temperature by day predicted means are not shown as no significant effect of storage temperature was found.

Table S2. The comparison in post incubation viability and recovery measured.

Day	Post Incubation Viability (%)	Post Incubation Recovery (%)
0	88.81 (81.25, 96.38)	53.05 (38.08, 67.48)
1	54.13 (46.99, 61.27)	23.31 (14.59, 35.11)
2	54.23 (47.09, 61.37)	32.24 (21.09, 45.86)
3	44.96 (36.99, 52.93)	16.09 (9.19, 26.65)
4	27.3 (17.57, 37.03)	4.95 (2.35, 10.12)

Data are predicted means (95%CI). Temperature by day predicted means are not shown as no significant effect of storage temperature was found.
